# Supplementary material for: Automated pipeline for EEG artifact reduction (APPEAR) recorded during fMRI
Source: J Neural Eng. Author manuscript; Available in PMC 2023 Dec 5. (PMC10696919; doi:10.1088/1741-2552/ac1037)
Supplement: Supplementary Material [file NIHMS1831827-supplement-Supplementary_Material.docx]

**Supplementary Material for:**

Automated Pipeline for EEG Artifact Reduction (APPEAR)

Recorded during fMRI

Ahmad Mayeli^1,2*^, Obada Al Zoubi^1,2*^, Kaylee Henry^1,3^, Chung Ki Wong^1^, Evan J. White^1^, Qingfei Luo^1^, Vadim Zotev^1^, Hazem Refai^2^, Tulsa 1000 Investigators^1#^, Jerzy Bodurka^1,4**^

^1^Laureate Institute for Brain Research, Tulsa, OK, United States

^2^Electrical and Computer Engineering, University of Oklahoma, Tulsa, OK, United States

^3^Department of Biomedical Engineering, University of Arkansas, Fayetteville, AR, United States

^4^Stephenson School of Biomedical Engineering, University of Oklahoma, Tulsa, OK, United States

*** Co-first Authors**

Ahmad Mayeli and Obada Al Zoubi

**** Correspondence:**Jerzy Bodurka
[jbodurka@laureateinstitute.org](mailto:jbodurka@laureateinstitute.org)

^#^The Tulsa 1000 Investigators include the following contributors: Robin Aupperle, Ph.D., Jerzy Bodurka, Ph.D., Justin Feinstein, Ph.D., Sahib S. Khalsa, M.D., Ph.D., Rayus Kuplicki, Ph.D., Martin P. Paulus, M.D., Jonathan Savitz, Ph.D., Jennifer Stewart, Ph.D., Teresa A. Victor, Ph.D.

**This file includes:**

1. Seventeen supplementary figures – pages 2 and 6-22
2. Details of detecting BCG components – pages 3-5
3. One supplementary table – page 23


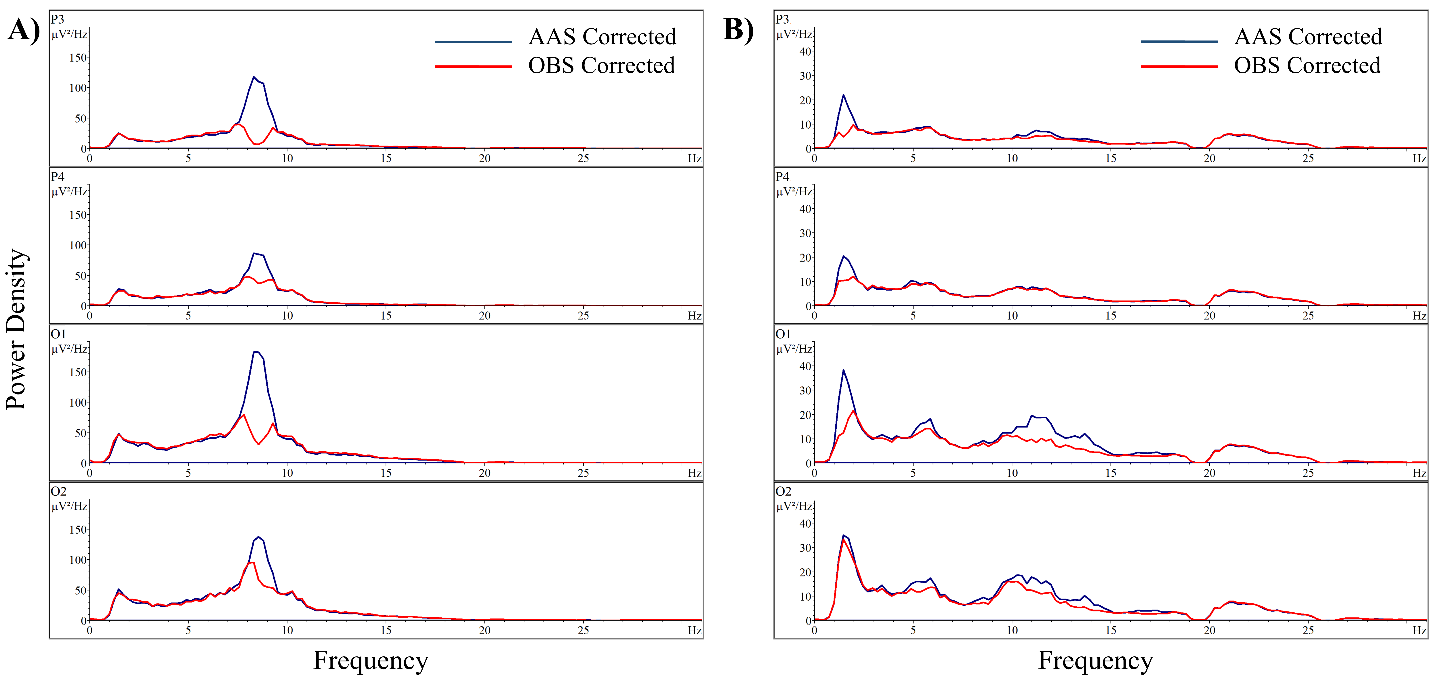
**Supplementary Figure S1:** Comparison between Power Spectral Density (PSD) after applying Average Artifact Subtraction (AAS, black line) and Optimal Basis Sets (OBS, red line) for subject 5 (**A**) and subject 8 (**B**).

**Supplementary Figure S1**

**Details for detecting independent components (IC) associated with ballistocardiogram (BCG) artifacts.**

The power spectrum is divided into two ranges, cardioballistic (2-7 Hz) and neuronal (8-12 Hz). If an IC has a cardioballistic artifact, the power spectrum shows peaks in both the cardioballistic and neuronal frequency ranges. The method used by (Wong et al, 2018) determines the rise of the peaks in both regions and requires that they meet four conditions to be considered as a BCG artifact. Condition (i) states that a large peak must be present in the cardioballistic frequency range; Condition (ii) states the Rise of the Neuronal peak (*R*_N_) must be small; and if (ii) is not satisfied, then Conditions (iii) and (iv) define comparable spectrum amplitudes required in the cardioballistic and neuronal ranges for a BCG IC. For Condition (ii), to obtain the full R_N_, a frequency range is defined between the Frequency at the Local Minimum (*f*_LMin_) immediately below 8 Hz and the Frequency at the Peak (*f*_P_) in the neuronal range. If such a local minimum exists, the frequency range is taken as [*f*_LMin_, *f*_P_]; otherwise the frequency range becomes [8 Hz, *f*_P_]. The power at the lower and upper bound of the frequency range is denoted as *S*(*f*) where *f*=*f*_LMin_ or *f*=8 Hz and *f*=*f*_P_, respectively. The *R*_N_ is calculated as the difference between the *S*(*f*_P_) and the minimum power given within the frequency range (either *S*(*f*_LMin_) or *S*(8 Hz)), calculated with either equation [S1] or [S2] depending on if a local minimum immediately below 8 Hz exists.

**[S1]** *R*_N_ = *S*(*f*_P_ ) - min(*S*(*f*), *f* ∈ [*f*_LMin_ *f*_P_]), if *f*_LMin_ exists

**[S2]** *R*_N_ =*S*(*f*_P_ ) - min(*S*(*f*), *f*∈[8 Hz, *f*_P_]) if *f*_LMin_ does not exist

For Conditions (iii) and (iv), the minimum power (*S*_min_) below the neuronal peak frequency is defined as a baseline for each spectrum. A cardioballistic motion IC is recognized when the average power (*S*_ave_) in the cardioballistic frequency range is comparable to the neuronal peak rise. There may be multiple Peaks in the CardioBallistic (*P*_cb_) range, *i*=1,…,*P*_cb_. Condition (iii) requires that the cardioballistic peaks have a local minimum on the left and a peak rise larger than 0.2*S*_ave_. Condition (iv) requires that the maximum peak Rise in the CardioBallistic range (*R*_cb_), or the average power over the cardioballistic range (*S*_cb_) is sufficiently large compared to the Rise of the Neuronal peak (*R*_N_). Condition (iv) is met if any of the following equations [S3 – S5] are met:

**[S3]** *R*_N_ <= 0.33 *S*_ave_

**[S4]** For *P*_cb_ cardioballistic peaks with local left minimum and *R*_cb,k_ > 0.2 *S*_ave_, where *k*=1,…, *P*_cb_, max({*R*_cb,k_}, *k*=1,…, *P*_cb_) > *R*_N_ -3

**[S5]** For *P*_cb_ cardioballistic peaks with local left minimum and *R*_cb,k_ > 0.2 *S*_ave_, where *k*=1,…, *P*_cb_, mean(*S*(*g*), *g* ∈ [2 Hz, 7 Hz]) - *S*_min_ >0.33 *R*_N_, max({*S*_cb,k_}, *k*=1,…, *P*_cb_) > *S*_N_ -3

where in [S5] the peak power of the neuronal range is stated as *S*_N_.

The spatial projection of each IC onto the EEG channel space forms a topographic map. The spatial projection vector is interpolated using the MATLAB function, griddata. Normally, BCG ICs exhibit bipolar topographies, i.e. opposite polarities for opposite regions (Zotev at al., 2012). During the topographic map analysis, the values are normalized. Then, two sets of polarity regions, primary and secondary, are defined. (Wong et al., 2018) creates polarity arc regions, defined as the overlapping polarity regions using a topographic map boundary with a width of 0.2. Any region not defined by the primary and secondary regions are labeled as neutral regions. Using the three conditions developed in (Wong et al, 2018), the BCG ICs are flagged. Condition (i) requires that there be up to one neutral region in the topographic map; Condition (ii) requires that only one positive (or negative) polarity region and polarity arc region are allowed in the topographic map; Condition (iii) ensures that there is a left/right opposite polarity region with one negative (or positive) primary polarity region and polarity arc region; and Condition (iv) sets the minimum areas for the secondary polarity region and polarity arc region in the topographic map.

In the time-series of a BCG IC, there are distinct peaks (approximately every 1 second) caused by cardiac pulsations. Removing the BCG IC from the EEG time-series signal shows a steady signal reduction at the pulsation peaks. Looking at the signal contribution of a BCG IC, the average positive and negative magnitudes (α_+_ and α_-_, respectively) of the reduced signal (α’) after removing the IC are compared to the original time-series signal (α). In (Wong et al, 2018), the thresholds for the average positive and negative magnitudes for any channel j are: (i) 0.5(α_j+_’/α_j+_ + α_j-_’/α_j-_) < 0.97 and (ii) min(α_j+_’/α_j+_ + α_j-_’/α_j-_) < 0.95. If these two thresholds are met, the IC is flagged as a BCG artifact.

**Supplementary Figure S2: The figure demonstrates the heuristic property of independent component analysis (ICA).** The time series of 10 seconds EEG data from one participant **(A)** and the data after randomly shuffled (the entire length of EEG data was shuffled, but we just present 10 seconds of the data for better visualization) **(B)** before running independent component analysis (ICA). The topography map of independent components (ICs) after running ICA on the original EEG data **(C)** and shuffled EEG data **(D)**. The artifactual components are marked (they are in black squares). **E)** The EEG data after removing artifactual ICs. **F)** The shuffled EEG data after removing artifactual ICs and reverting to the original sort order (re-ordering was performed after running inverse ICA). Although the order of ICs is different when we applied ICA on the original EEG and shuffled one, in both, we could distinguish the artifactual ICs, and after removing them and reverting the shuffled data to the original sort order, we get the same artifact-reduced results.


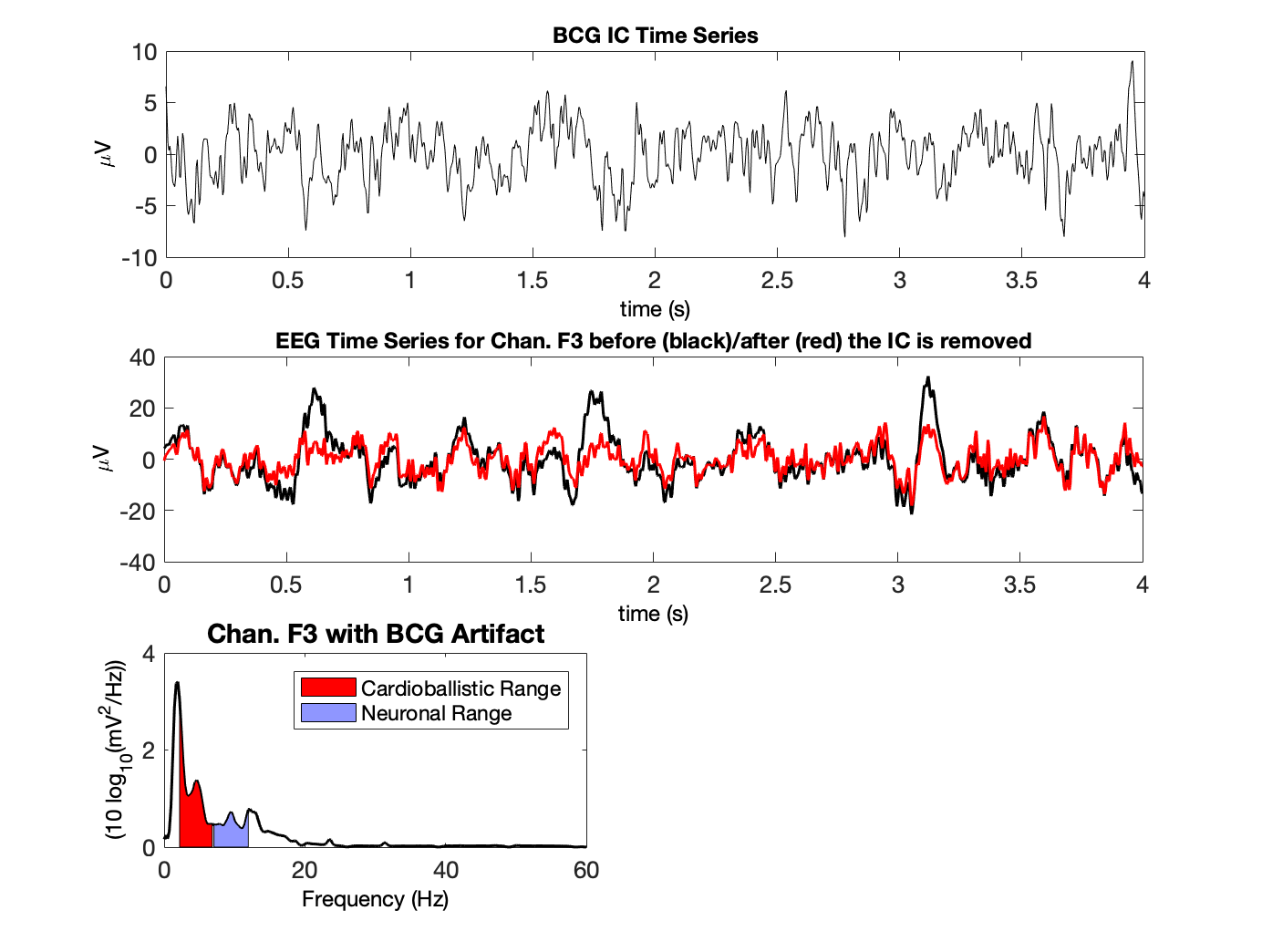

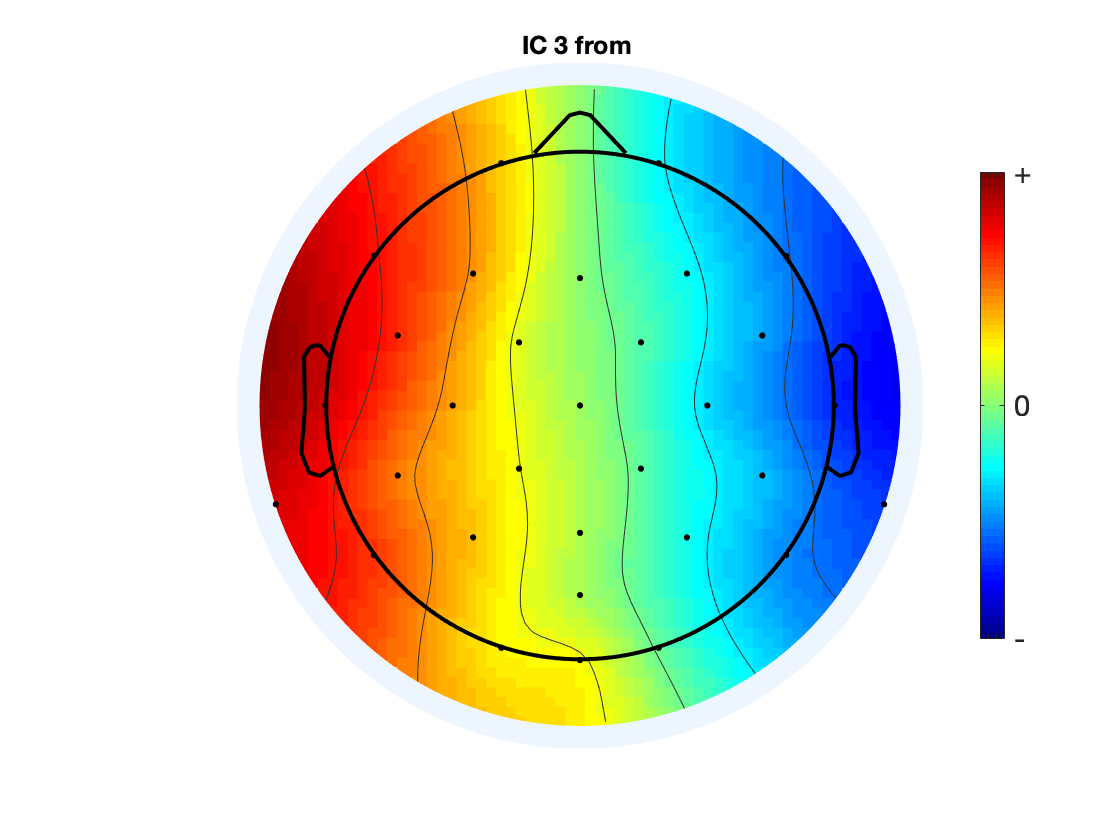


**Supplementary Figure S2**

**Supplementary Figure S3:** An example of a Ballistocardiogram (BCG) component.


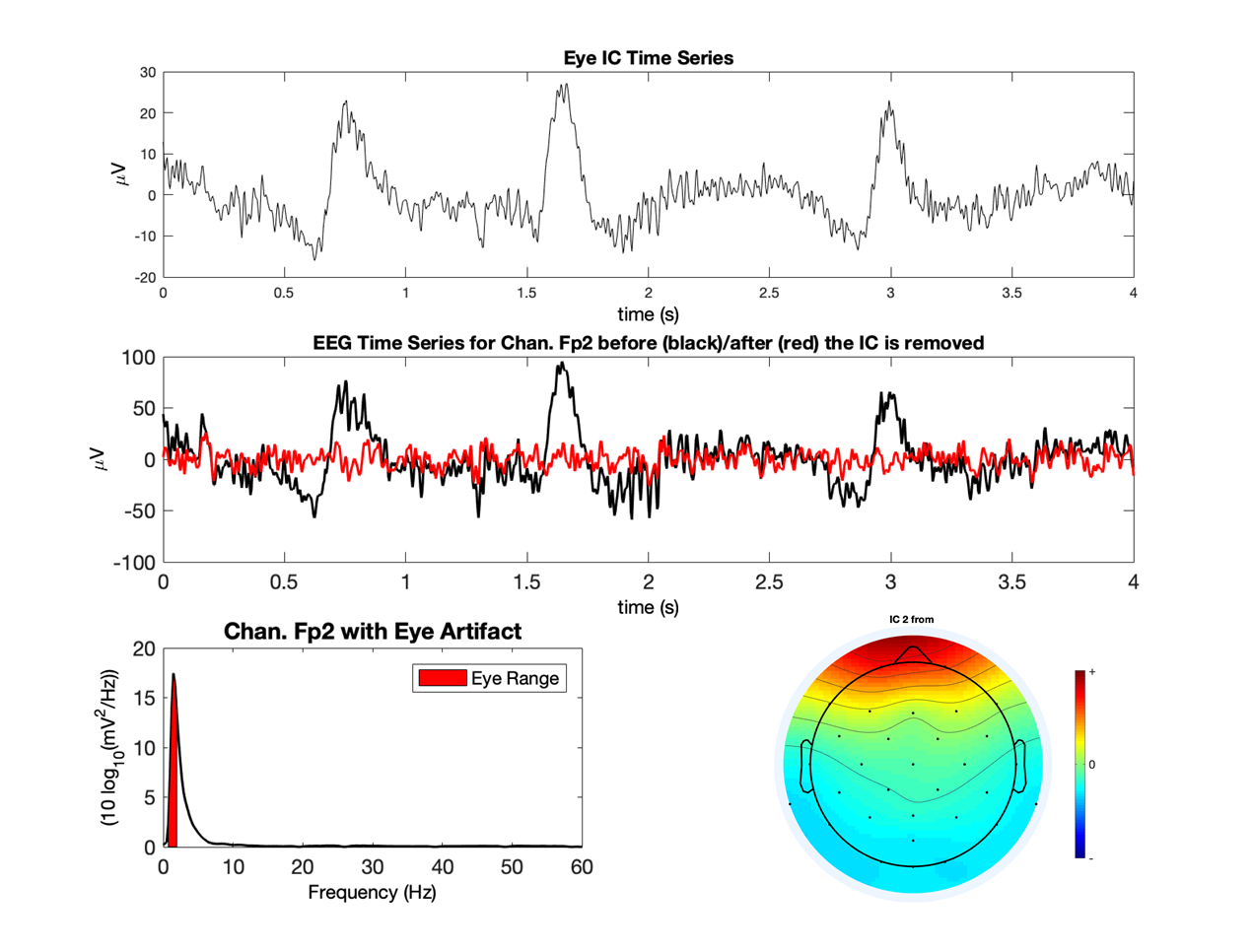


**Supplementary Figure S3**

**Supplementary Figure S4:** An example of a Blink Component. Blink ICs can be identified by their strong spatial projection in the frontal area and low frequency activity in delta band.

**Supplementary Figure S4**


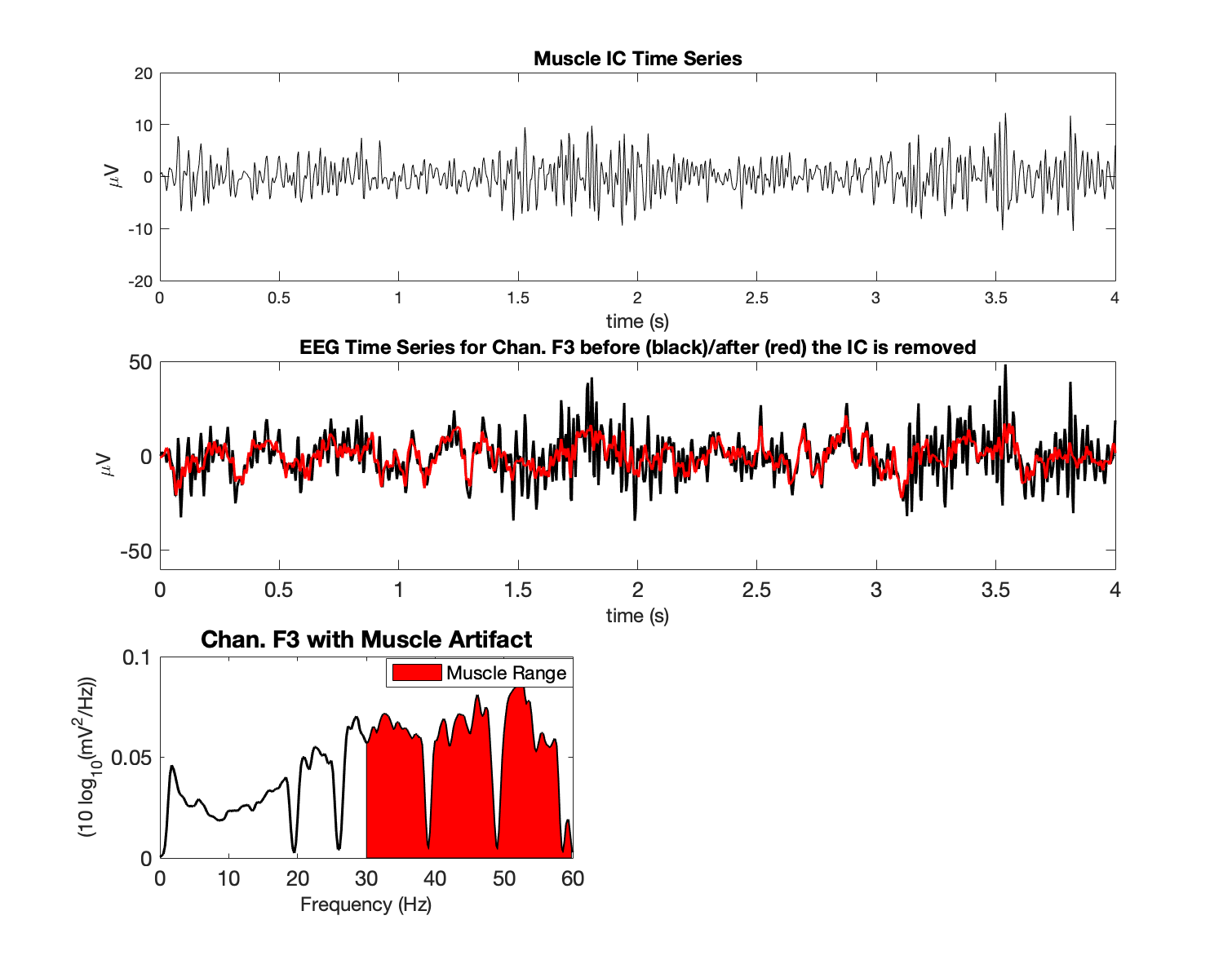

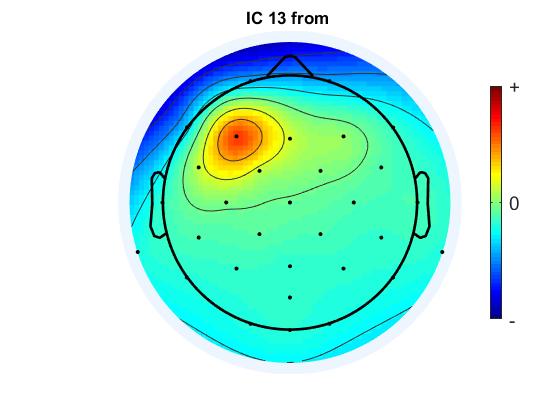


**Supplementary Figure S5:** An example of a Muscle Component. Muscle electrical activity or “electromyogenic” (EMG) artifacts exhibit widespread high-frequency activity due to asynchronous motor action units. These components are flagged if the power of the signal is spread out in frequencies higher than 30 Hz, known as the gamma band

**
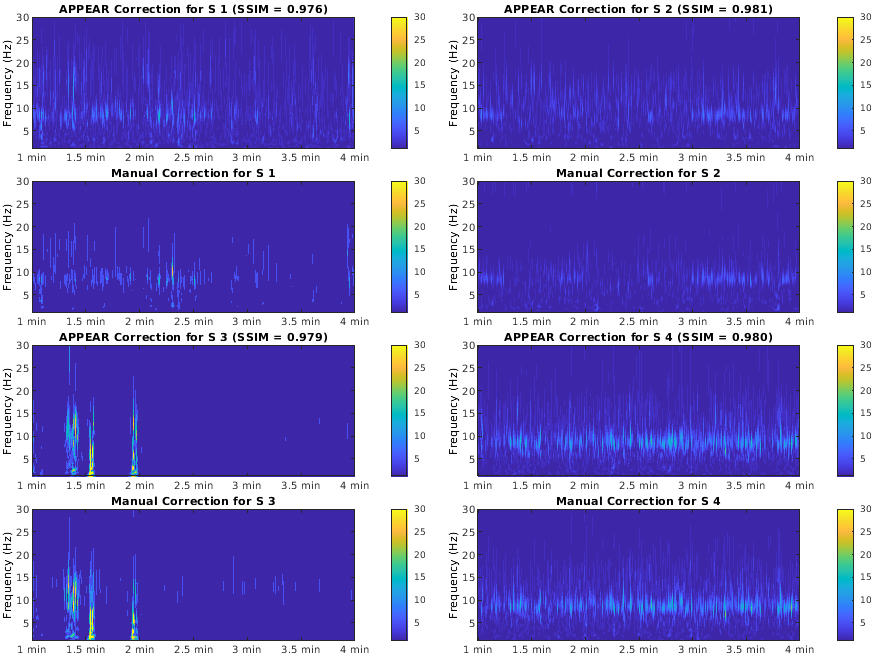
**

**Supplementary Figure S6:** A comparison between CWT results from APPEAR and manually corrected data for subjects 1 through 4.

**
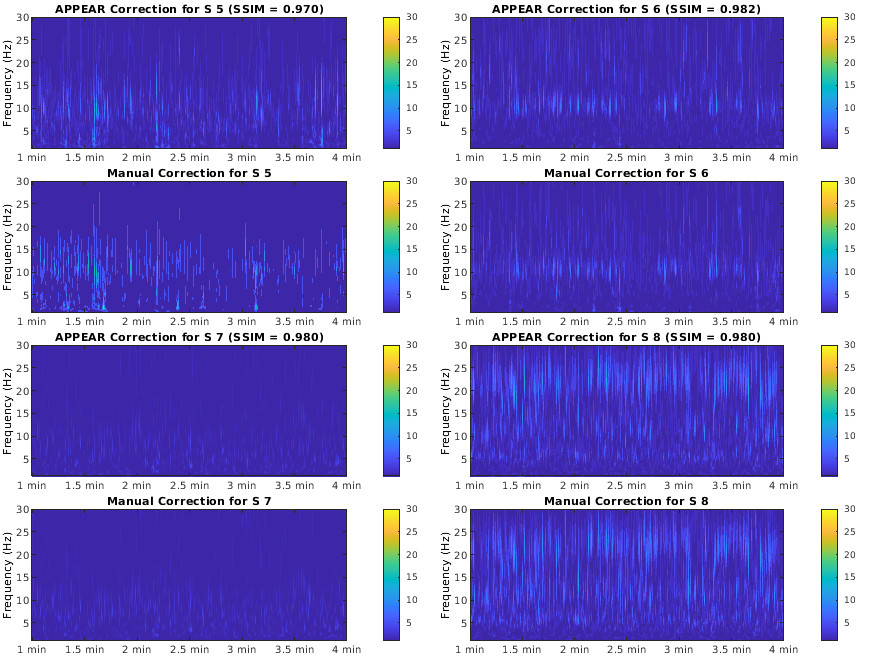
**

**S****upplementary Figure S7:** A comparison between CWT results from APPEAR and manually corrected data for subjects 5 through 8.

**
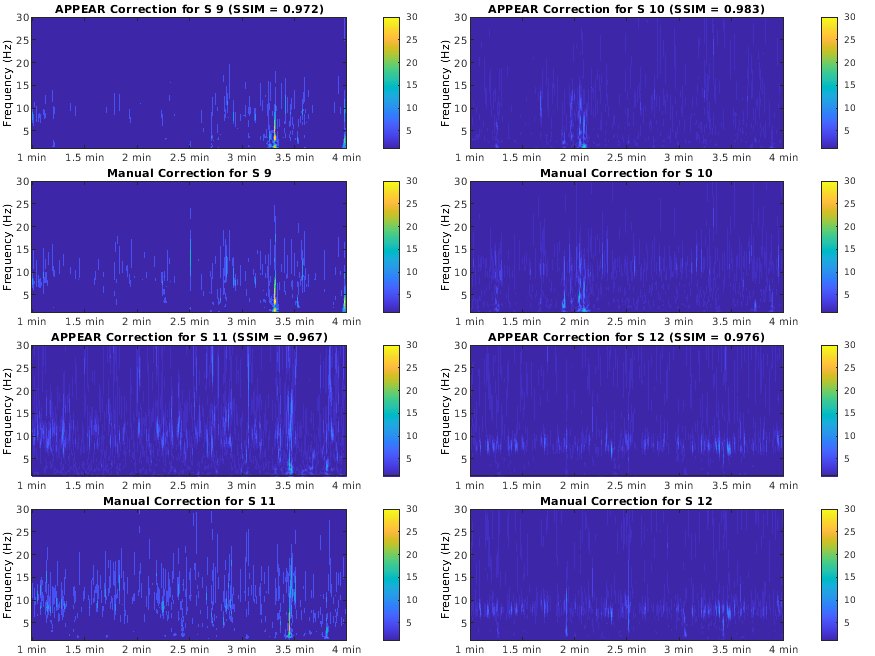
**

**Supplementary Figure S8:** A comparison between CWT results from APPEAR and manually corrected data for subjects 9 through 12.

**
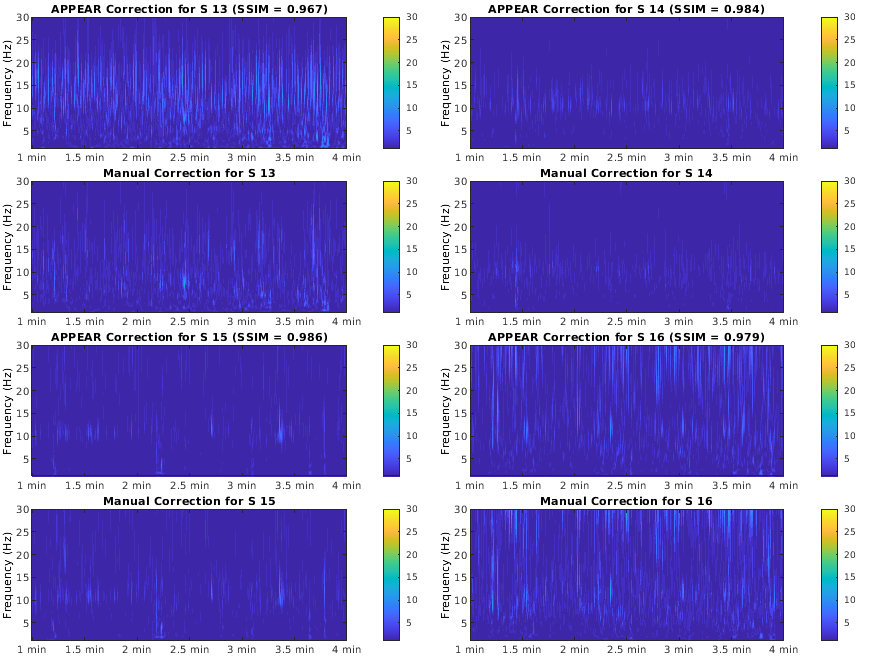
**

**Supplementary Figure S9:** A comparison between CWT results from APPEAR and manually corrected data for subjects 13 through 16.


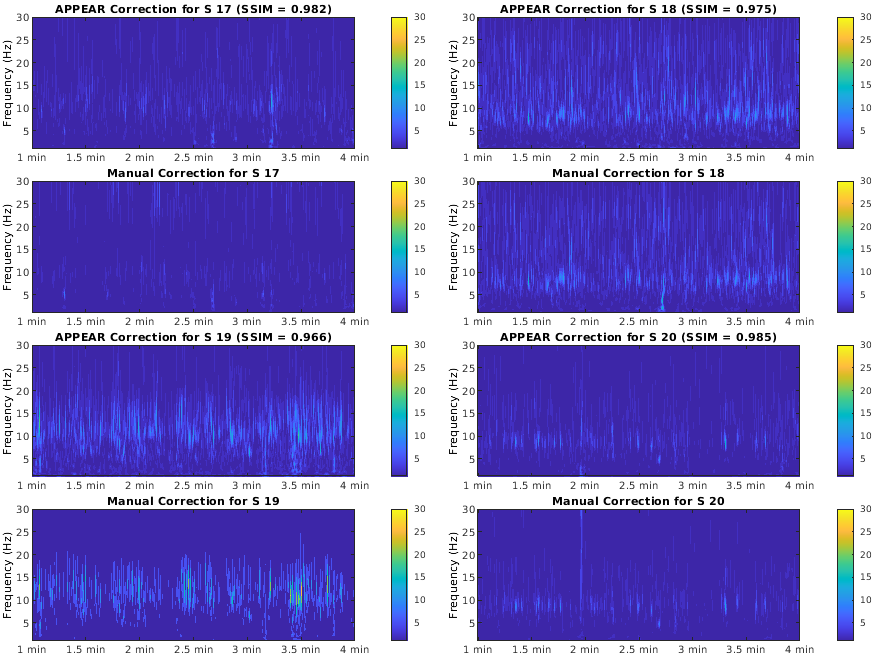


**Supplementary Figure S10:** A comparison between CWT results from APPEAR and manually corrected data for subjects 17 through 20.


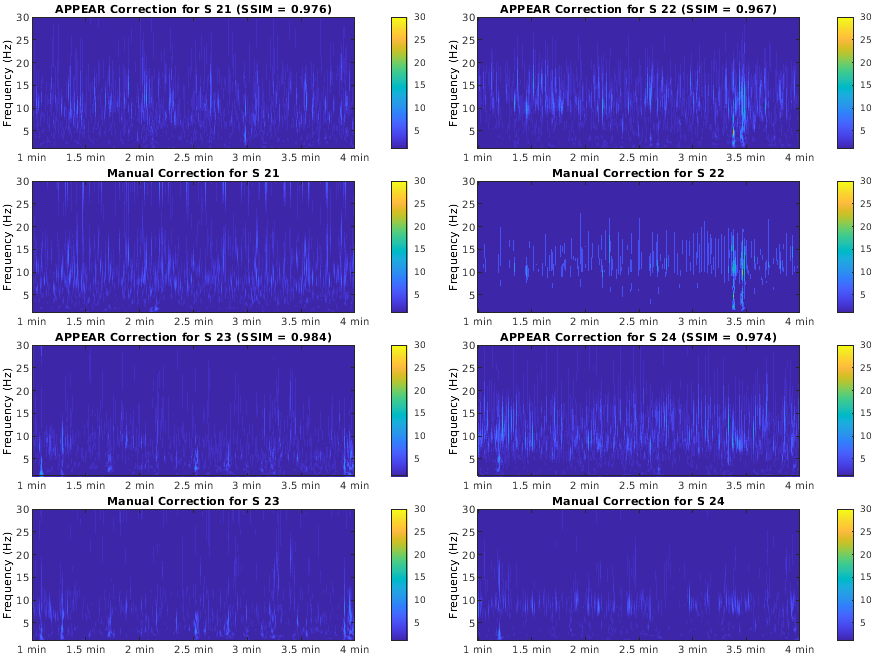


**Supplementary Figure S11:** A comparison between CWT results from APPEAR and manually corrected data for subjects 21 through 24.


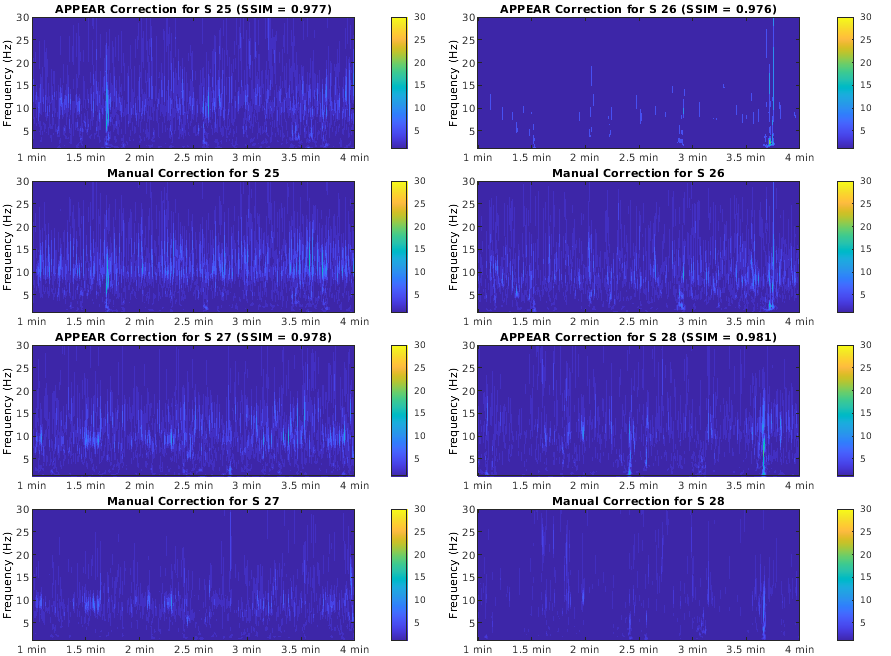


**Supplementary Figure S12:** A comparison between CWT results from APPEAR and manually corrected data for subjects 25 through 28.

**
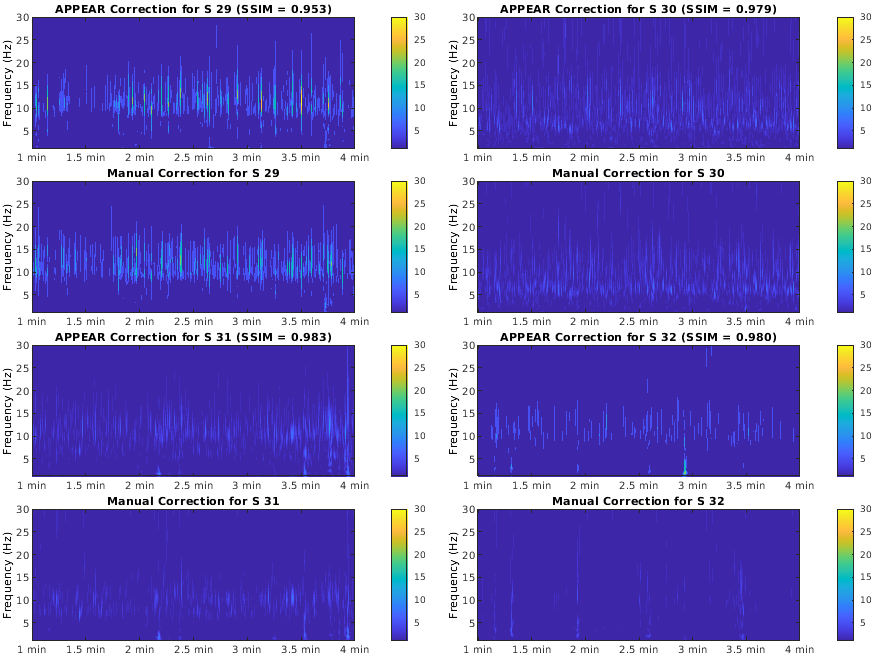
**

**Supplementary Figure S13:** A comparison between CWT results from APPEAR and manually corrected data for subjects 29 through 32.

**
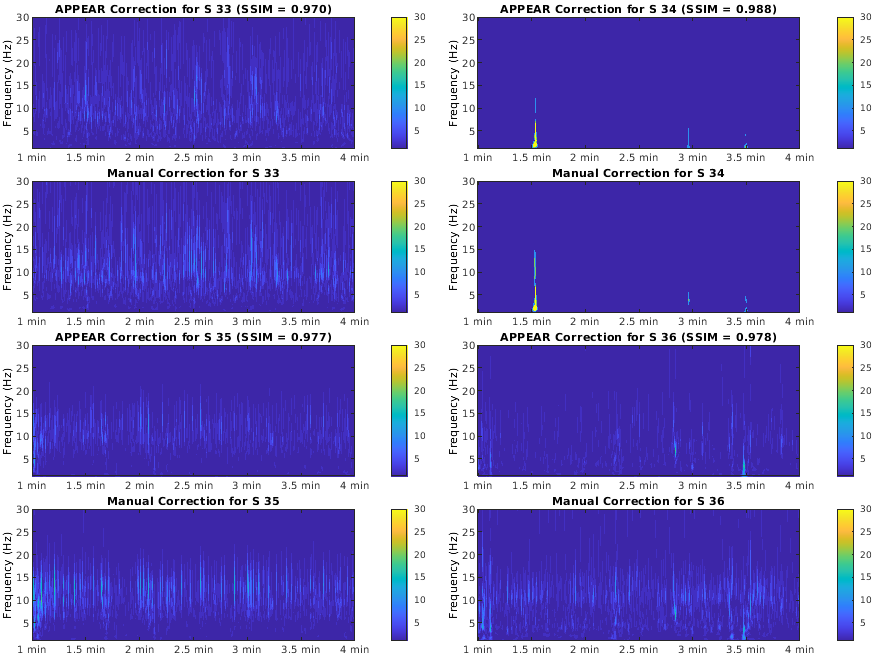
**

**Supplementary Figure S14:** A comparison between CWT results from APPEAR and manually corrected data for subjects 33 through 36.


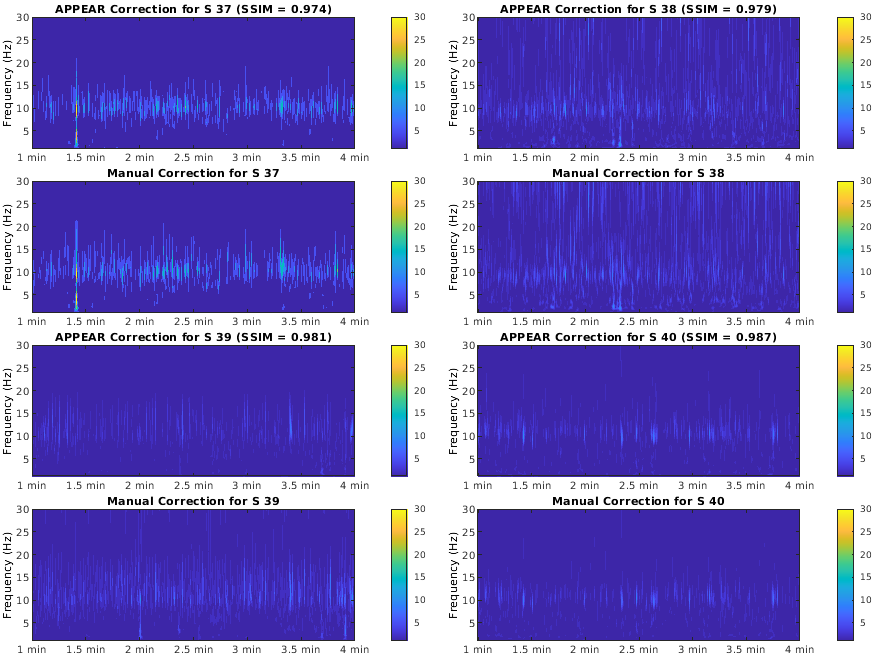


**Supplementary Figure S15:** A comparison between CWT results from APPEAR and manually corrected data for subjects 37 through 40.

**
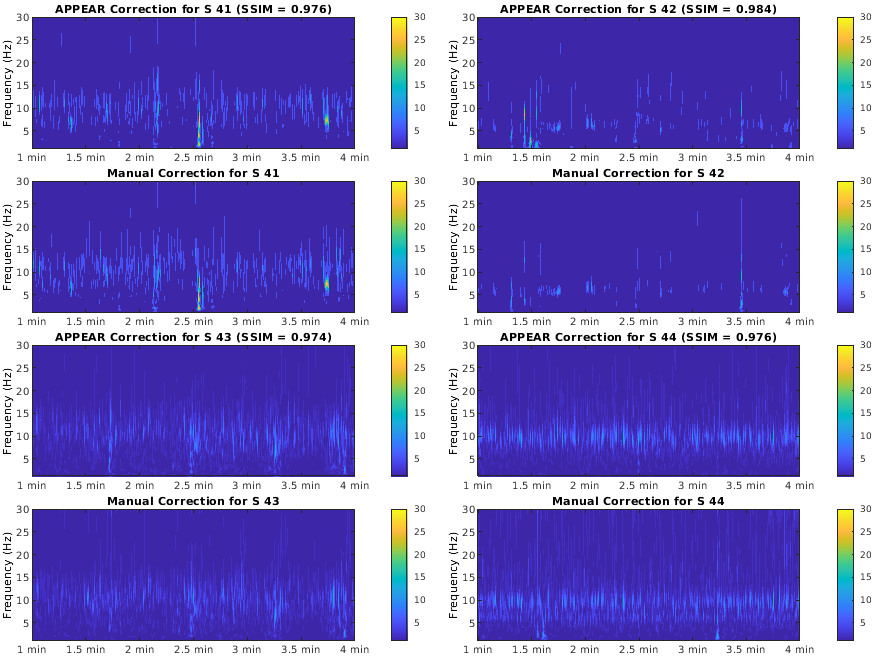
**

**Supplementary Figure S16:** A comparison between CWT results from APPEAR and manually corrected data for subjects 41 through 44.

**
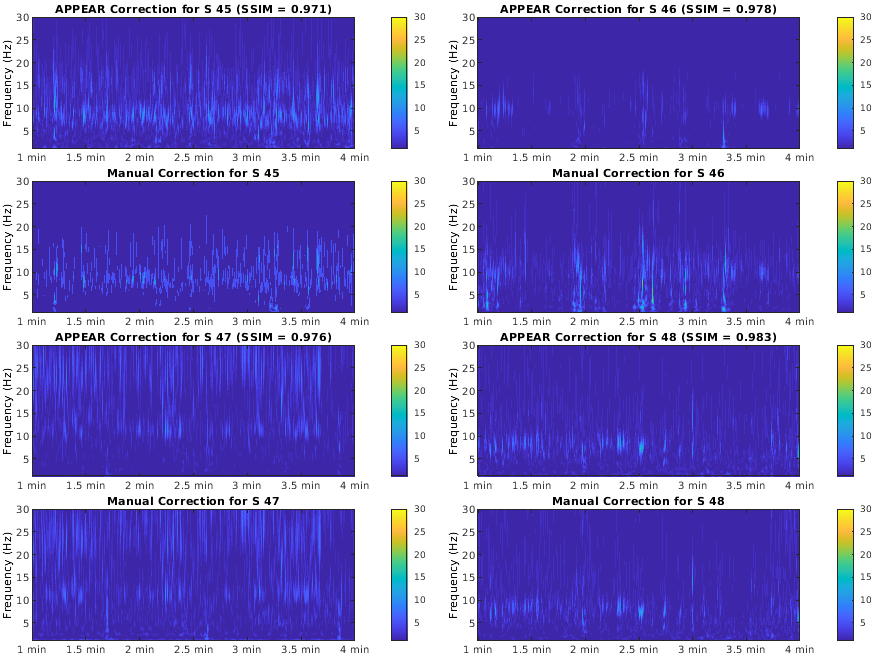
**

**Supplementary Figure S17:** A comparison between CWT results from APPEAR and manually corrected data for subjects 45 through 48.

**Supplementary Table S1.** The average heart rate for all subjects (Rest and Stop Signal experiments) derived from: i) the simultaneous ECG signal recorded with EEG (calculated with the FMRIB plug-in implemented in MATLAB); ii) EEG data (computed using independent component analysis); iii) physiological pulse oximetry signal concurrently and independently recorded during EEG-fMRI (MATLAB peak detection function - findpeaks). The values in parentheses show the absolute heart rate difference measured between ECG/EEG and pulse oximetry.

| **Rest** | **Subject1** | **Subject2** | **Subject3** | **Subject4** | **Subject5** | **Subject6** | **Subject7** | **Subject8** |
| --- | --- | --- | --- | --- | --- | --- | --- | --- |
| ECG (FMRIB) | 62.79 (0.38) | 62.86 (0.42) | 66.27 (0.19) | 72.62 (0.09) | 69.59 (0.02) | 79.46 (0.18) | 76.00 (0.05) | 44.98 (0.08) |
| EEG (ICA-Based) | 63.18 (0.02) | 117.83 (54.55) | 66.44 (0.02) | 72.56 (0.04) | 69.53 (0.07) | 79.56 (0.27) | 76.03 (0.03) | 45.03 (0.03) |
| Pulse oximetry (peak detection) | 63.17 | 63.28 | 66.46 | 72.52 | 69.61 | 79.29 | 76.06 | 45.06 |
| **Stop Signal** |  |  |  |  |  |  |  |  |
| ECG (FMRIB) | 66.58 (0.09) | 68.93 (4.36) | 65.44 (0.92) | 75.80 (0.06) | 76.99 (0.09) | 85.01 (0.523) | 77.31 (2.040) | 43.86 (0.17) |
| EEG (ICA-Based) | 66.07 (0.41) | 64.45 (0.12) | 64.49 (0.02) | 76.33 (0.47) | 76.87 (0.03) | 85.08 (0.60) | 75.46 (0.19) | 44.00 (0.02) |
| Pulse oximetry (peak detection) | 66.49 | 64.57 | 64.52 | 75.86 | 76.90 | 84.49 | 75.27 | 44.03 |
